# The application of in silico experimental model in the assessment of ciprofloxacin and levofloxacin interaction with main SARS-CoV-2 targets: S-, E- and TMPRSS2 proteins, RNA-dependent RNA polymerase and papain-like protease (PLpro)—preliminary molecular docking analysis

**DOI:** 10.1007/s43440-021-00282-8

**Published:** 2021-05-30

**Authors:** Krzysztof Marciniec, Artur Beberok, Stanisław Boryczka, Dorota Wrześniok

**Affiliations:** 1 Department of Organic Chemistry, Faculty of Pharmaceutical Sciences in Sosnowiec, Medical University of Silesia, Jagiellońska 4, 41-200 Sosnowiec, Poland; 2 Department of Pharmaceutical Chemistry, Faculty of Pharmaceutical Sciences in Sosnowiec, Medical University of Silesia, Jagiellońska 4, 41-200 Sosnowiec, Poland

**Keywords:** Fluoroquinolones, E protein, S protein, TMPRSS2 protein, RNA-dependent RNA polymerase, Papain-like protease (PL^PRO^)

## Abstract

**Background:**

The new severe acute respiratory syndrome coronavirus 2 (SARS-CoV-2) was identified at the end of 2019. Despite growing understanding of SARS-CoV-2 in virology as well as many molecular studies, except remdesivir, no specific anti-SARS-CoV-2 drug has been officially approved.

**Methods:**

In the present study molecular docking technique was applied to test binding affinity of ciprofloxacin and levofloxacin—two commercially available fluoroquinolones, to SARS-CoV-2 S-, E- and TMPRSS2 proteins, RNA-dependent RNA polymerase and papain-like protease (PL^PRO^). Chloroquine and dexamethasone were used as reference positive controls.

**Results:**

When analyzing the molecular docking data it was noticed that ciprofloxacin and levofloxacin possess lower binding energy with S protein as compared to the references. In the case of TMPRSS2 protein and PL^PRO^ protease the best docked ligand was levofloxacin and in the case of E proteins and RNA-dependent RNA polymerase the best docked ligands were levofloxacin and dexamethasone. Moreover, a molecular dynamics study also reveals that ciprofloxacin and levofloxacin form a stable complex with E- and TMPRSS2 proteins, RNA polymerase and papain-like protease (PL^PRO^).

**Conclusions:**

The revealed data indicate that ciprofloxacin and levofloxacin could interact and potentially inhibit crucial SARS-CoV-2 proteins.

## Introduction

The coronavirus disease 2019 (COVID-19) pandemic caused by SARS-CoV-2 poses a huge challenge to medicine. The effective therapeutic methods are urgently needed to treat the infection. Developing new drug is a time consuming process. Thus, drug repositioning seems to be reasonable strategy to overcome the disease [1, 2]. It was reported that the main drug targets for SARS-CoV-2 may include S-, E- and TMPRSS2, Mpro proteins [3–5], RNA-dependent RNA polymerase (RdRp) [6] and papain-like protease (PLpro) [7]. Therefore an in silico approach of screening existing database to find a variety of compounds that could inhibit SARS-CoV-2 molecular targets can save time by filtering out compounds worthy of both in vitro as well as in vivo analysis. Vijayakumar et al. [8] have demonstrated that natural flavonoids and synthetic indole chalcones may exert antiviral effect by the potential interaction with essential proteins of SARS-CoV-2. Analysis of protein–ligand docking data revealed that (1) cyanidin may suppress RdRp, (2) genistein, kaempferol and quercetin or arbutin could interact with S-proteins, (3) quercetin displayed strong interactions on SARS-CoV-2 Mpro protein. Moreover, quercetin was pointed as an exceptional compound for further in vitro and in vivo studies. The docking and simulation analysis conducted by Gentile et al. [3] showed that chloroquine and hydroxychloroquine influenced the functionality of E protein due to interactions that modify the flexibility of the protein structure. In turn, Dey et al. [9] as well as Tomar and Arkin [10] revealed that tretinoin, mefenamic acid, ondansetron and gliclazide, memantine could consist potential inhibitors of ion channels formed by SARS-CoV-2 E protein. Wu et al. [11] have conducted computational analysis of therapeutic targets for SARS-CoV-2 such as S protein, RdRp and PLpro proteases. They found that (1) ribavirin, doxycycline, tigecycline or sulfasalazine in the case of PLpro, (2) chlorhexidine, cefuroxime, cortisone or sylybin in the case of RdRp, (3) prazosin, itraconazole, penicillin or dabigatran in the case of S protein, may be considered as potential inhibitors and thus possible new strategies for drug repositioning to treat SARS-CoV-2 infections. In addition previous research efforts to develop antiviral agents against members of Coronaviridae family pointing that the Angiotensin-converting enzyme II (ACE2) entry receptor may consist proper drug target [7]. Although the new promising inhibitors targeting ACE2 were characterized they did not advance clinically due to significant side effects [12].

Fluoroquinolones (e.g. ciprofloxacin, levofloxacin), synthetic antibiotics widely used to treat bacterial infections, were shown to exert antiviral, antifungal, and antiparasitic activity. What is important, these effects were seen at the clinically achievable concentrations and may result from one common mode of action: the inhibition of type II topoisomerases or inhibition of viral helicases [13]. Previously we have demonstrated for the first time that ciprofloxacin and moxifloxacin may interact with COVID-19 Main Protease (M^pro^) pointing the basis for further in vitro as well as in vivo analysis underlying the two already-marketed fluoroquinolones derivatives repositioning to treat SARS-CoV-2 infections [14]. In this study, we used molecular docking strategies to repurpose fluoroquinolone derivatives—ciprofloxacin and levofloxacin for COVID-19.

Docking results suggest potential COVID-19 inhibitors: ciprofloxacin and levofloxacin were docked onto the COVID-19 proteins with a lower negative dock energy value. The best recorded binding energy value was obtained for ciprofloxacin-S protein complex. On the other hand, consistent results indicate that levofloxacin binds strongly to the E- and PL^PRO^ proteins and RNA-dependent RNA polymerase. This work thus highlights the possibility of exploring fluoroquinolone antibiotics as a potential main SARS-CoV-2 proteins blockers, which could be an important therapeutic strategy in the treatment of coronaviruses. Moreover, the obtained results consist the basis for further studies towards these drugs in vitro as well as in vivo inhibitory potential and prepare for clinical trial applications.

## Materials and methods

The three-dimensional structures of ciprofloxacin, levofloxacin and references were optimized and energy minimized using Gaussian 16 (rev. A.03) computer code [15] using the density functional theory (DFT, B3LYP) and 63–11 + G(d,p) basis sets. A selected SARS-CoV-2 proteins for molecular docking studies were obtained from the Protein Data Bank (https://www.rcsb.org/). We used crystal structure of the SARS-Cov-2 RNA-dependent RNA polymerase (PDB ID: 6M71), SARS-CoV-2 spike ectodomain (PDB ID: 6VYB) and papain-like protease of SARS-CoV-2 (PDB ID: 6W9C). The homology models of the SARS-CoV-2 E protein catalytic domain and Transmembrane Serine Protease 2 were generated using SWISS-MODEL server [16].

AutoDock Vina [17] tool compiled in PyRx [18] was used for the docking analysis. The region of interest used for AutoDock Vina docking was defined as *X* = 114,416, *Y* = 122.286, *Z* = 139.457 for RdRp, *X* = − 0.496, *Y* = 0.00, *Z* = 0.00 for E protein, *X* = 177,973, *Y* = 198.333, *Z* = 227.722 Å for S protein, *X* = − 33,784, *Y* = 20,933, *Z* = 33,306 for papain-like protease, and *X* = 10,507, *Y* = − 3557, *Z* = 30,903 for Transmembrane Serine Protease 2. The volume was set as 40 × 40 × 40 Å. After calculations, only the 9 highest-scored poses were returned as a docking result for ligand-cavity configuration. All the obtained results were presented in kcal/mol. Molecular docking details were visualized using the BIOVIA Discovery Studio virtual environment [19].

Based on docking results, the lowest energy and best-posed complexes were selected for the molecular dynamics (MD) simulation using Nanoscale Molecular Dynamics software ver. 2.13 (NAMD, https://www.ks.uiuc.edu/Research/namd/) [20]. All files were generated using visual molecular dynamics (VMD) [21]. The parameters of ligands for the CHARMM/CGenFF force field [22] were obtained from the CGenFF server (https://cgenff.umaryland.edu). The parameterized ligands were inserted into the protein and saved in the form of a protein–ligand complex by user-friendly software, QwikMD [23] with the binding pocket residues, and then the protein–ligand complex was immersed in the center of a box of water molecules with a TIP3P water box. 0.15 M ions (Na+ and Cl–) were added to provide charge neutralization and electrostatic screening. CHARMM (Chemistry at HARvard Macromolecular Mechanics) 36 parameter file for proteins and lipids; phi and psi cross-term map correction were used in the force field for proteins with similar chemical structures. For the minimization and equilibration of the complexes in the water box, we assumed force-field parameters excluding scaling of 1.0 Å. All atoms, including those of hydrogen, were illustrated explicitly. Complexes preliminary energy was minimized via 2000 steps at constant temperature (310 K), followed by simulation of an additional 144,000 steps with Langevin dynamics to control the kinetic energy, temperature, and/or pressure of the system. Finally, the solvated protein–ligand complex system was equilibrated with 500,000 minimization steps, and 5,000,000 runs for 10 ns. The root-mean-square deviations were analyzed using Visual Molecular Dynamics package (VMD, https://www.ks.uiuc.edu/Research/vmd/) [21]. The MolAICal [24] is used to calculate the MM/GBSA between ligands and EGFR protein based on molecular dynamical (MD) simulated results by NAMD.

## Results

Ciprofloxacin and levofloxacin can each exist as three chemical species: cationic, anionic, and zwitterionic depending on the pH of the aqueous solution. For ciprofloxacin, the experimentally measured p*K*_a1_ and p*K*_a2_ values are 5.9 and 8.2 [25], and for the levofloxacin experimentally measured p*K*_a1_ and p*K*_a2_ values are 5.5 and 8.0, respectively [26].

**Fig. 1:**
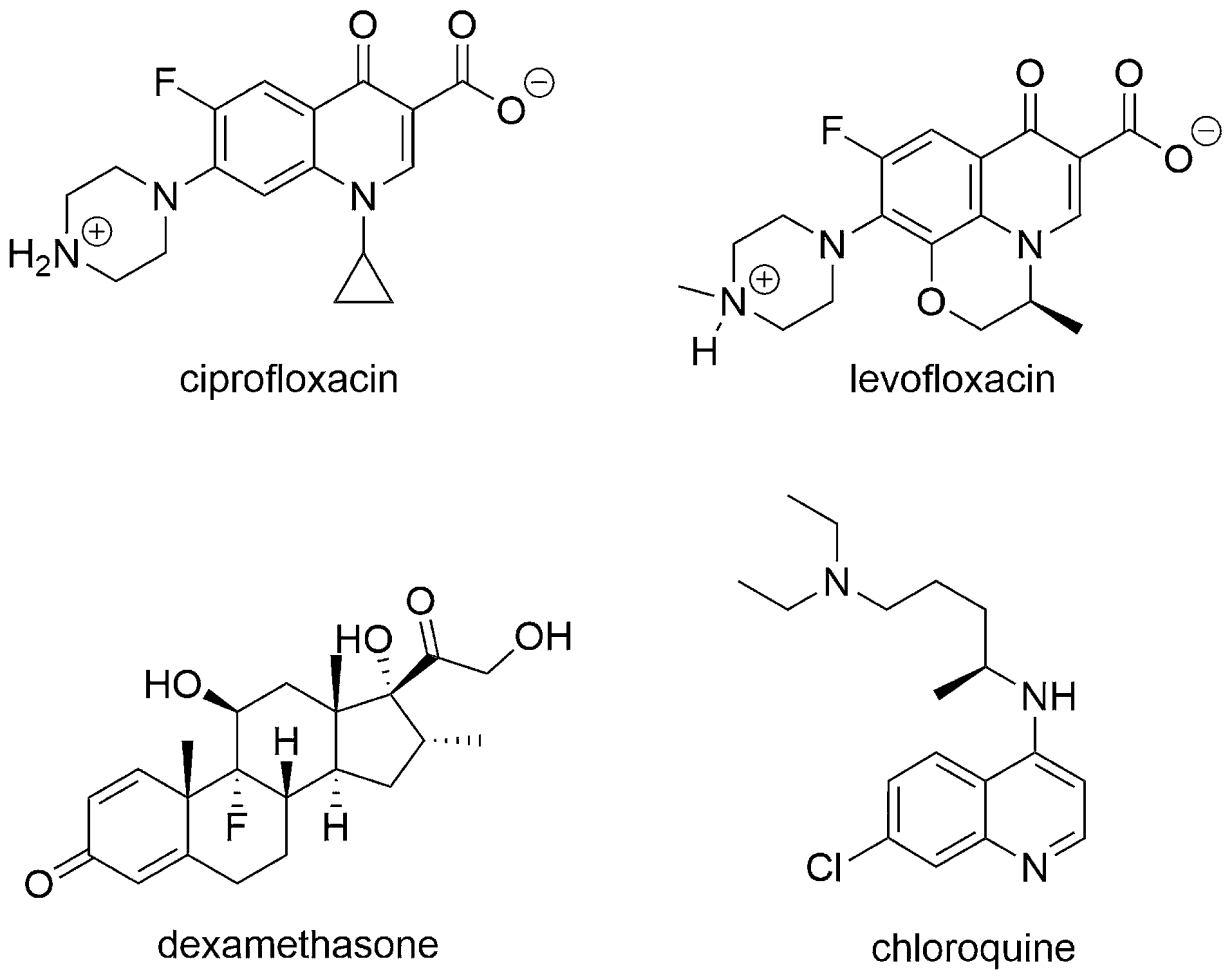
Structure of compounds used in this study

The structures of the zwitterionic states of ciprofloxacin and levofloxacin used in docking calculations are presented in Fig. 1. All of the ligand molecules were 3D optimized and energy minimized using Gaussian 16 computer code [15].

In this study, we used the AutoDock Vina program (referred to as Vina) in in silico research. Vina is a turnkey computational docking program based on a simple scoring function and rapid gradient-optimization conformational search [17]. All the data of docking score are shown in Table 1. The lowest scores of binding energy (kcal/mol) of complexes reflect to the strongest binding affinity, and the most probable ligand–protein system in vivo. Moreover, we additionally calculated the values of *K*_*i*_. The *K*_*i*_ is reflective of the binding affinity and the IC_50_ is more reflective of the functional strength of the inhibitor for a drug. Since the *K*_*i*_ takes into account the IC_50_ is its calculation, the *K*_*i*_ in being reported more often by drug companies. It is readily apparent that the relationship of IC_50_ to *K*_*i*_ is dependent upon the type of inhibition and the mechanism of the reaction. In some enzymatic reactions at a specific substrate concentration *K*_*i*_ does not equal IC_50_, when competitive inhibition kinetics apply; however, *K*_*i*_ is equal to IC_50_, under the conditions of either noncompetitive or uncompetitive kinetics [27]. Calculation of the *K*_*i*_ from the binding energy of the pose generated by Vina was performed using the following equations:

$$\square G/\left( {R \times T} \right) = \ln K_{i} \, ({\text{for }}T = 298\,{\text{K and }}R = 1.987\,{\text{kcal}} \times {\text{K}}^{ - 1} \times {\text{mol}}^{ - 1} )$$

and next:

$$K_{i} = \exp \left[ {\square G/\left( {R \times T} \right)} \right].$$

**Table 1:** Scoring functions ∆G [kcal × mol^−1^] and p*K*_I_ values of tested compounds

Ligand	Protein									
	S protein		TMPRSS2		RdRp		PLpro		E protein	
	∆*G*	p*K*_I_	∆*G*	p*K*_I_	∆*G*	p*K*_I_	∆*G*	p*K*_I_	∆*G*	p*K*_I_
Ciprofloxacin	− 6.8	4.99	− 6.5	4.77	− 6.3	4.62	− 6.1	4.48	− 6.7	4.92
Levofloxacin	− 6.6	4.84	− 7.4	5.43	− 6.8	4.99	− 6.8	4.99	− 7.2	5.28
Dexamethasone	− 6.4	4.69	− 6.6	4.84	− 6.8	4.99	− 6.5	4.77	− 7.2	5.28
Chloroquine	− 5.2	3.81	− 5.7	4.18	− 6.3	4.62	− 5.3	3.89	− 6.3	4.62

The binding of the S1 subunit of the spike protein (S protein) to a host cell receptor via the receptor binding domain (RBD) of the S1 subunit is crucial for SARS-CoV entry into the host cell. The S1 subunit is also divided into domain A and B. It has been shown that SARS-CoV-2 S glycoprotein binds favorably to the human Angiotensin-converting enzyme 2 (hACE2) receptor via the S1B. Residues 331–524 of the S glycoprotein as the receptor binding domain of the spike have recently been identified [28].

**Fig. 2:**
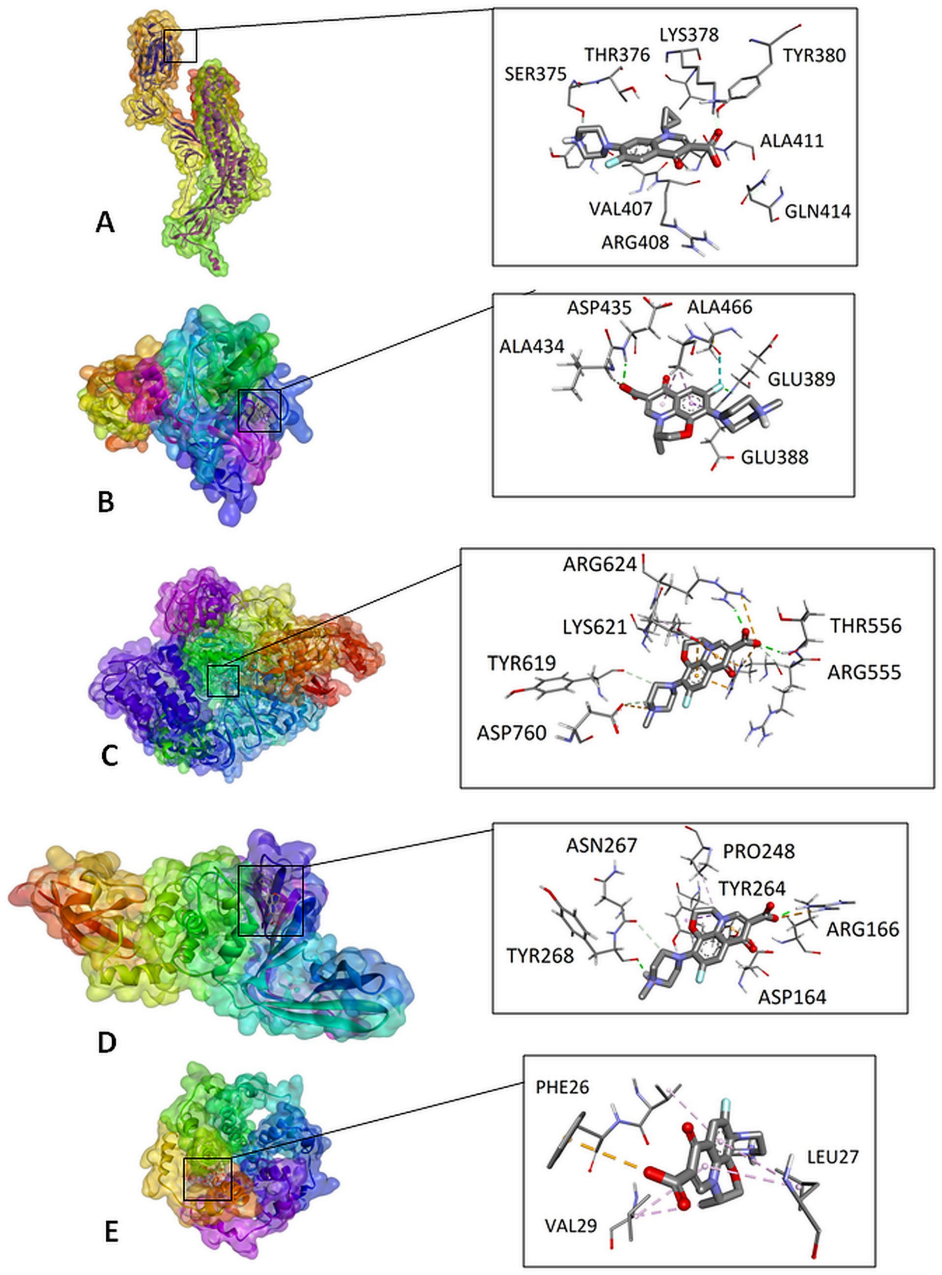
The lowest-energy docking poses of S protein complex with ciprofloxacin (**A**), TMPRSS2 protein with ciprofloxacin (**B**), RdRp with levofloxacin (**C**), PLpro with levofloxacin (**D**), and E protein with levofloxacin (**E**)

**Fig. 3:**
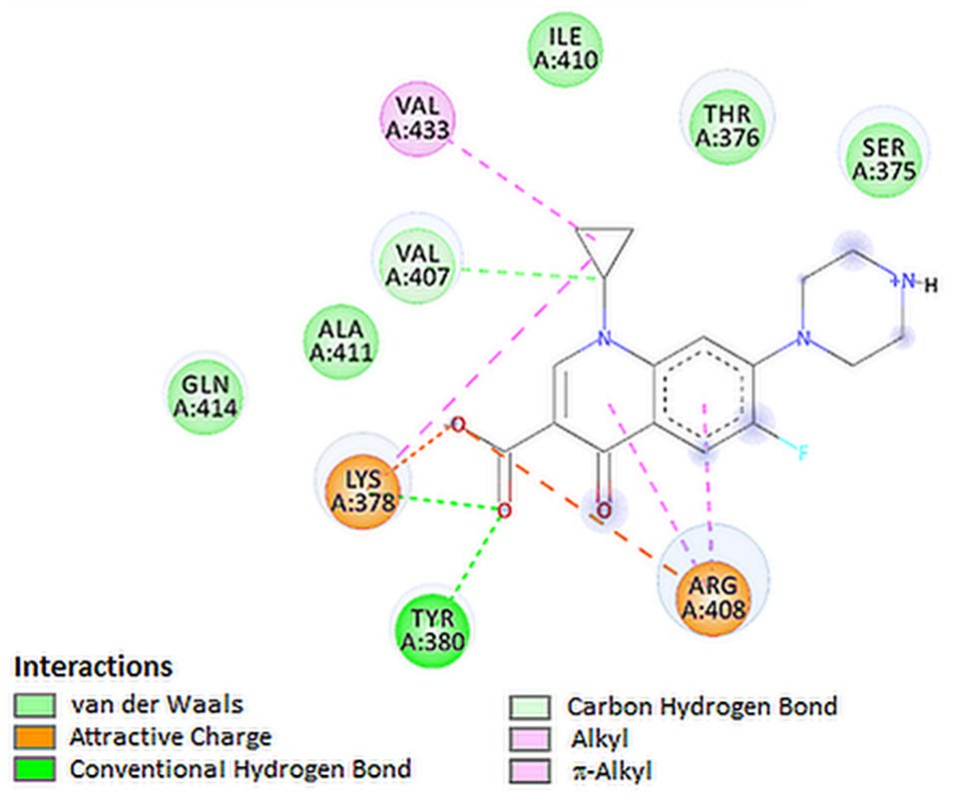
2D visualization of interaction between ciprofloxacin and S protein

Results obtained in the Vina program indicate that ciprofloxacin showed lower binding energy with S protein compared to the references (Table 1). Figures 2A and 3 present the possible interaction of ciprofloxacin inside the binding pocket S protein. Corresponding amino acids that are significantly involved in the hydrophobic interactions are as follows: Val407, Lys378, Val433 and Arg408. Strong hydrogen bond interaction between Lys378 and Tyr380 and carboxylate group increase the stability of the ligand–receptor complex. Ciprofloxacin formed, with the active site of S protein, a network of attractive charge bonds with Lys378 and Arg408 residues (Table 3).

Transmembrane protease serine subfamily member 2 (TMPRSS2) was found to be the host protein that resides on the cell membrane. It mediates the entering of pathogenic human coronaviruses into cells by cleaving as well as activating the viral Spike (S) protein. It was shown, that TMPRSS2 could be co-expressed in lung tissue with angiotensin converting enzyme 2 (ACE2) that acts as the cell surface receptor for SARS and SARS-CoV-2 [29].

Molecular dynamics (MD) calculation provide a better understanding of structure–function relationships in motion and other conformational changes by the proteins. Therefore, an MD study was conducted in this paper to evaluate the dynamic behavior of the top-scoring complexes. The Molecular Mechanics/Generalized Born Surface Area (MM/GBSA) method was selected for rescoring complexes because it is the fastest force field-based method that computes the free energy of binding, as compared to the other computational free energy methods, such as free energy perturbation (FEP) or thermodynamic integration (TI) methods. Comparison studies have also shown that MM/GBSA outperforms Molecular Mechanics/Poisson Boltzmann Surface Area MM/PBSA [30]. The MM/GBSA calculation was performed using MolAICal software [24]. MolAICal provides a way to calculate the MM/GBSA based on the output results of molecular dynamics simulations that are carried out by NAMD [20]. The calculated binding free energies are shown in Table 2.

**Table 2:** Calculated binding free energies of tested compounds [kcal/mol]

Complex	Δ*G*	Δ*E*_(internal)_	Δ*E*_(elektrost.)_ + Δ*G*_(sol.)_	Δ*E*_(VDW)_ + Δ*G*_(sol.)_
S protein/ciprofloxacin	− 10.35	− 1.54 × 10^−6^	2.95	− 13.30
TMPRSS2/ciprofloxacin	− 13.98	− 1.07 × 10^−6^	7.96	− 21.94
RdRp/levofloxacin	− 6.27	− 4.20 × 10^−7^	8.55	− 14.80
PLpro/levofloxacin	− 11.14	− 1.35 × 10^−5^	6.42	− 17.57
E protein/levofloxacin	− 21.72	− 3.45 × 10^−5^	10.85	− 32.58

The binding free energies of tested complexes range from − 21.72 to − 6.27 kcal/mol. According to the calculations performed, E protein–levofloxacin complex is most stable compared to the other complexes. Analyzing the complexes ligand root-mean-square deviation (RMSD), root-mean-square fluctuation (RMSF), and hydrogen bonding can provide insights into a structural conformation faced by the protein and ligand during simulation.

**Fig. 4:**
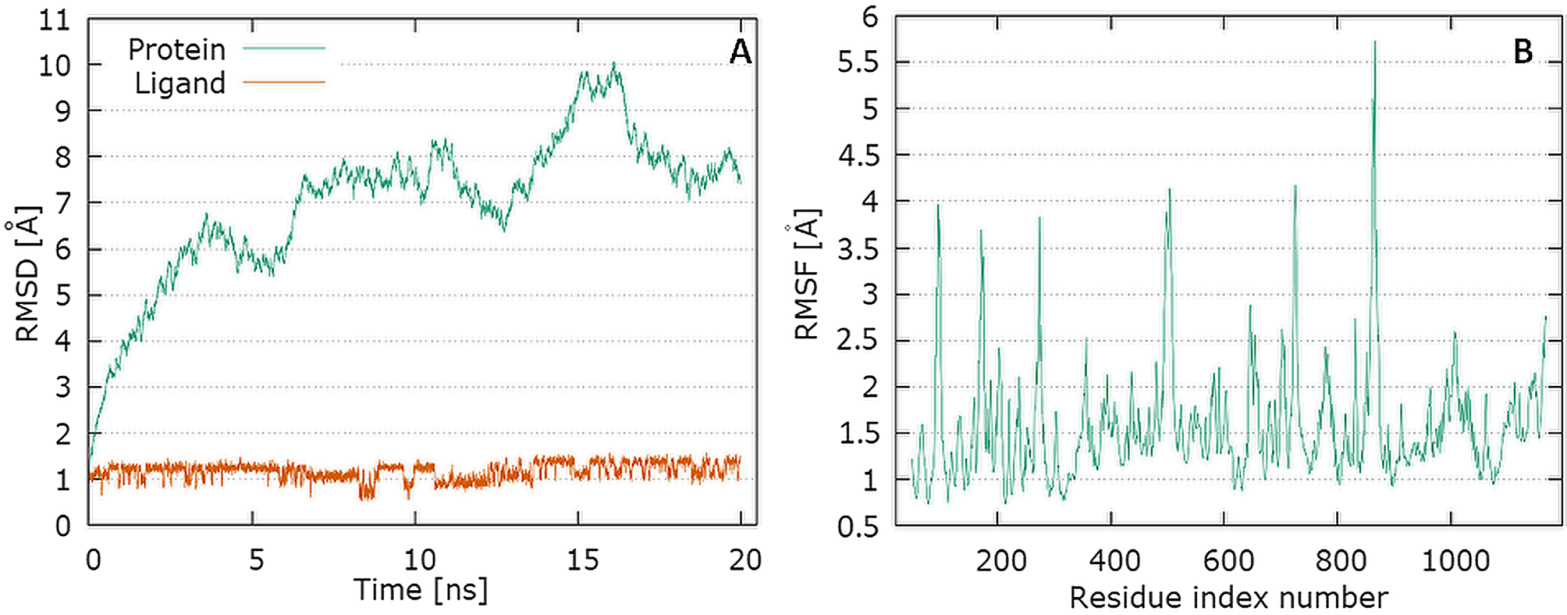
Graphical representation of the plots showing protein CαRMSD [Å] versus time (20 ns) (**A**) and protein RMSF [Å] versus residue index number (**B**) for ciprofloxacin complex of S protein

**Fig. 5:**
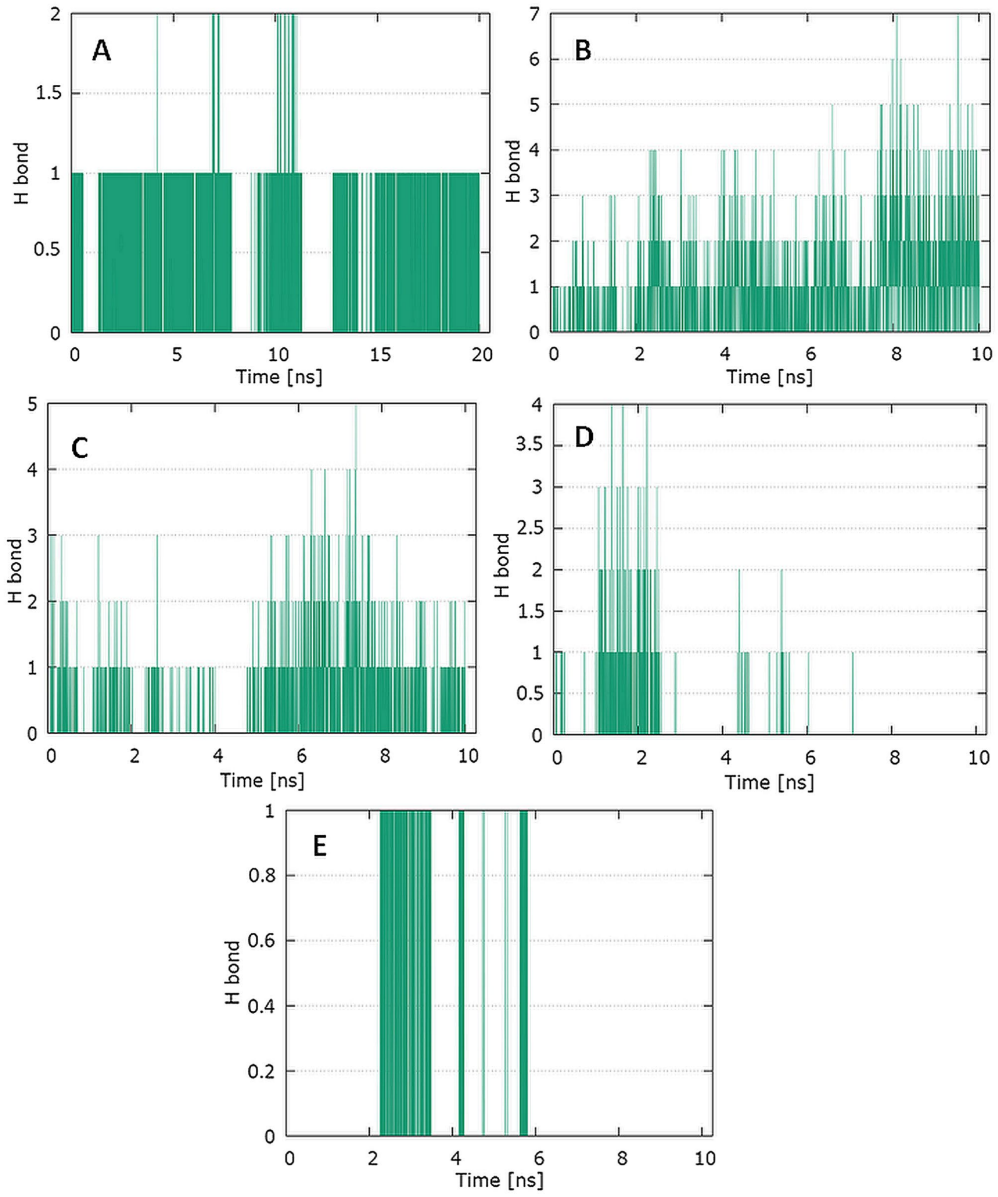
Pictorial representation of the number of H-bond contacts formed by ciprofloxacin with S protein **(A),** ciprofloxacin with TMPRSS2 protein (**B**), levofloxacin with RdRp (**C**), levofloxacin with PLpro (**D**), and levofloxacin with E protein (**E**)

The plots for S protein Cα versus time for the simulation with ciprofloxacin are shown in Fig. 4A. The visual analysis of the trajectory confirms the stability of the structure of protein at around 6 ns with peak at around 16 ns. The plot for ligand RMSD versus time shows a plateau value of 1.3 Å for the ciprofloxacin at around 0.2 ns. There was no a significant shift in RMSD values for the ligand backbone. The deviation value observed was within limits, which is quite stable for binding of the ligand with the S protein. The plot for protein RMSF versus residue number index shown in Fig. 4B describes fluctuations in the range of 0.5‒6 Å for complex S protein with ciprofloxacin. The catalytic residues are no longer higher then 1 Å and is suitable for stable binding. The data presented in Fig. 5 show the results of analysis of hydrogen bond contacts in the studied complexes. Ciprofloxacin forms two hydrogen bonds (Fig. 5A), and the binding with Lys378 residue occurs in the complex at the 18% time of measurement.

**Fig. 6:**
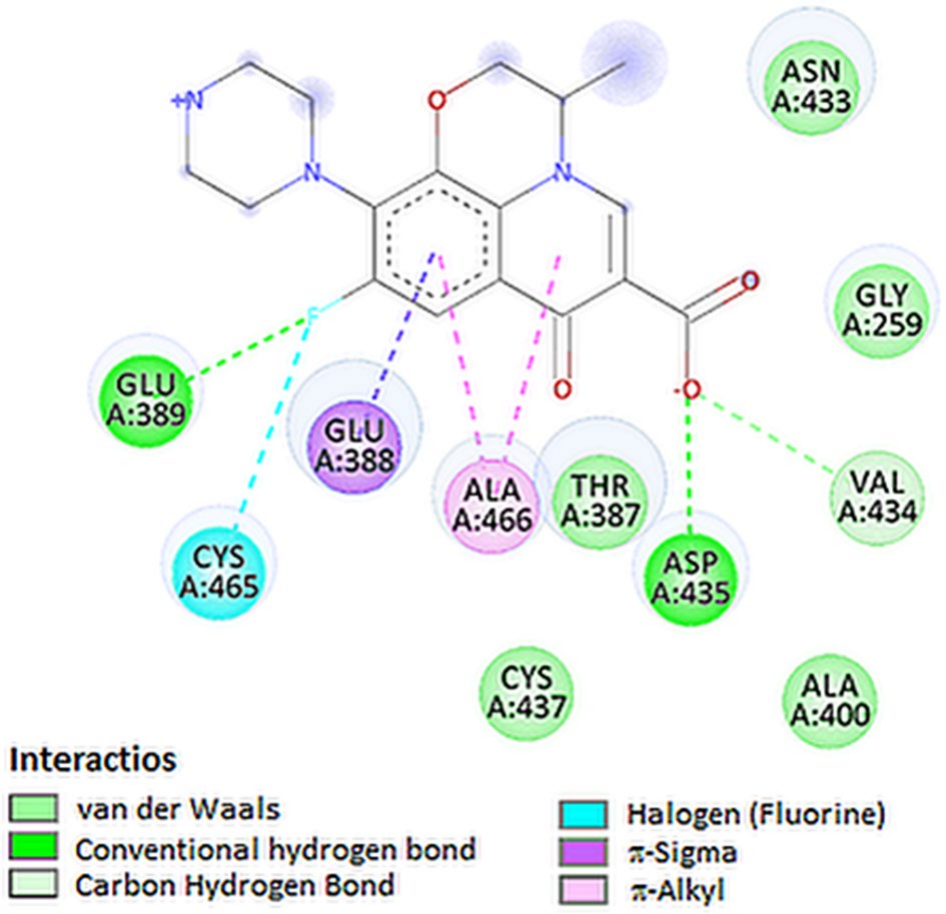
2D visualization of interaction between levofloxacin and TMPRSS2 protein

The best docked ligands to TMPRSS2 protein was levofloxacin. The top docked pose of levofloxacin in the binding site showed hydrophobic interactions with Val434, Cys465, Glu388 and Ala466 (Table 3, Figs. 2B and 6).

**Table 3:** Interaction of tested compounds with COVID-19 proteins

Protein		Ligand		Interaction	
Name	Residue	Name	Residue	Type	Distance (Å)
S protein	Lys378	Ciprofloxacin	Carboxylate	Attractive charge	4.75
	Arg408		Carboxylate	Attractive charge	5.46
	Lys378		Carboxylate	Conventional hydrogen bond	1.81
	Tyr380		Carboxylate	Conventional hydrogen bond	2.11
	Val407		Cyclopropyl	Carbon hydrogen bond	3.62
	Lys378		Cyclopropyl	Alkyl-alkyl	4.75
	Val433		Cyclopropyl	Alkyl-alkyl	4.33
	Arg408		Benzene ring	π-Alkyl	4.60
	Arg408		Pyridone ring	π-Alkyl	4.72
	Lys378	Levofloxacin	Carboxylate	Attractive charge	4.71
	Lys378		Carboxylate	Conventional hydrogen bond	1.96
	Tyr380		Carboxylate	Conventional hydrogen bond	2.47
	Tyr380		Pyridone oxygen	Conventional hydrogen bond	2.03
	Lys378		Pyridone ring	π-Cation	4.37
	Arg408		Benzene ring	π-Alkyl	4.58
	Arg408		Pyridone ring	π-Alkyl	4.52
	Phe543	Dexamethasone	Hydroxyl group	Conventional hydrogen bond	2.72
	Asn544		Hydroxyl group	Conventional hydrogen bond	2.91
	Leu390		Methyl group	Alkyl-alkyl	5.25
	Leu517		Pentene ring	Alkyl-alkyl	4.65
	Leu517		Methyl group	Alkyl-alkyl	5.31
	Phe464	Chloroquine	Isopropyl	π-Sigma	3.69
	Pro426		Chlorine atom	Alkyl-alkyl	4.41
	Pro426		Benzene ring	π-Alkyl	5.37
	Pro426		Pyridine ring	π-Alkyl	4.33
TMPRSS2	Asn398	Ciprofloxacin	Carboxylate	Conventional hydrogen bond	2.11
	Asp435		Pyridone ring	Carbon hydrogen bond	3.37
	Cys437		Cyclopropyl	Alkyl-alkyl	4.56
	Cys465		Cyclopropyl	Alkyl-alkyl	4.57
	Ala466		Cyclopropyl	Alkyl-alkyl	3.69
	Glu389	Levofloxacin	Fluorine	Conventional hydrogen bond	2.12
	Asp435		Carboxylate	Conventional hydrogen bond	2.23
	Val434		Carboxylate	Carbon hydrogen bond	2.62
	Cys465		Fluorine	Dipole-dipole	2.94
	Glu388		Benzene ring	π-Sigma	2.76
	Ala466		Benzene ring	π-Alkyl	3.97
	Ala466		Pyridone ring	π-Alkyl	3.44
	Gly462	Dexamethasone	Hydroxyl group	Conventional hydrogen bond	2.90
	Gln438		Hydroxyl group	Carbon hydrogen bond	2.45
	Gly462		Fluorine	Dipole-dipole	3.06
	Cys465		Methyl group	Alkyl-alkyl	4.78
	Asn433	Chloroquine	II° amine group	Conventional hydrogen bond	2.17
	Asp435		Ethyl	Carbon hydrogen bond	3.63
	Ala386		Ethyl	Carbon hydrogen bond	3.75
	Ala466		Pyridine ring	π-Sigma	3.36
	Cys437		Pyridine ring	π-Sulfur	5.55
	Cys465		Pyridine ring	π-Sulfur	5.88
	Ala466		Benzene ring	π-Alkyl	4.02
RdRp	Cys622	Ciprofloxacin	Pyridone ring	Conventional hydrogen bond	2.71
	Thr680		Carboxylate	Conventional hydrogen bond	2.75
	Thr687		Carboxylate	Conventional hydrogen bond	2.16
	Asn691		Pyridone ring	Conventional hydrogen bond	2.38
	Ser682		Carboxylate	Carbon hydrogen bond	2.71
	Tyr619		Fluorine	Dipole-dipole	3.52
	Asp623		Pyridone ring	π-Anion	3.52
	Asp623		Benzene ring	π-Anion	3.79
	Cys622		Pyridone ring	π-Sulfur	5.88
	Cys622		Benzene ring	π-Sulfur	5.57
	Arg553	Levofloxacin	Carboxylate	Salt bridge	2.56
	Arg624		Carboxylate	Attractive charge	4.33
	Asp760		Piperazine nitrogen	Attractive charge	4.69
	Thr556		Carboxylate	Conventional hydrogen bond	2.17
	Arg624		Carboxylate	Conventional hydrogen bond	2.06
	Arg555		Carboxylate	Carbon hydrogen bond	2.68
	Asp623		Morpholine ring	Carbon hydrogen bond	3.43
	Tyr619		Pyridone ring	Carbon hydrogen bond	3.41
	Asp760		Piperazine ring	Carbon hydrogen bond	3.44
	Arg553		Pyridone ring	π-Cation	3.28
	Arg553		Benzene ring	π-Cation	3.581
	Asp623		Pyridone ring	π-Anion	.52
	Asp623		Benzene ring	π-Anion	3.08
	Lys621		Morpholine ring	Alkyl-alkyl	3.89
	Asp760	Dexamethasone	Hydroxyl group	Conventional hydrogen bond	1.91
	Ser759		Carbonyl oxygen atom	Conventional hydrogen bond	2.24
	Asp623		Hydroxyl group	Conventional hydrogen bond	1.87
	Cys622		Carbonyl oxygen atom	Conventional hydrogen bond	2.10
	Lys621		Hydroxyl group	Conventional hydrogen bond	2.77
	Lys621		Carbonyl oxygen atom	Conventional hydrogen bond	2.88
	Ala688		Carbonyl oxygen atom	Carbon hydrogen bond	2.56
	Pro620		Hydroxyl group	Carbon hydrogen bond	2.49
	Asp760		Fluorine	Dipole-dipole	2.90
	Thr409	Chloroquine	Ethyl	Carbon hydrogen bond	3.43
	Tyr546		Benzene ring	π–π stacked	4.64
	Tyr546		Pyridine ring	π–π stacked	3.67
	Lys411		Chlorine	Halogen bond	3.55
	Lys411		Benzene ring	π-Alkyl	5.09
PLpro	Asp164	Ciprofloxacin	Piperazine nitrogen	Salt bridge	2.32
	Asp302		Piperazine nitrogen	Attractive charge	5.25
	Tyr268		Pyridone	Carbon hydrogen bond	3.31
	Tyr264		Benzene ring	π–π stacked	5.24
	Leu162		Pyridone ring	Alkyl	5.07
	Leu162		Cyclopropyl	π-Alkyl	5.49
	Arg166	Levofloxacin	Carboxylate	Attractive charge	3.82
	Tyr268		Piperazine nitrogen	Conventional hydrogen bond	1.84
	Asn267		Piperazine ring	Carbon hydrogen bond	2.13
	Asp164		Pyridone ring	π-Anion	3.47
	Tyr264		Methyl	π-Sigma	3.34
	Pro248		Methyl	Alkyl-alkyl	3.63
	Tyr273		Methyl	π-Alkyl	4.63
	Tyr264	Dexamethasone	Methyl group	π-Alkyl	4.83
	Pro248		Methyl group	Alkyl-alkyl	4.62
	Tyr264	Chloroquine	Pyridine ring	π–π stacked	3.73
	Tyr264		Benzene ring	π–π stacked	4.72
	Leu162		Chlorine	Halogen	4.97
	Pro248		Pyridine ring	π-Alkyl	5.15
E protein	Ile46	Ciprofloxacin	Piperazine nitrogen	Conventional hydrogen bond	2.08
	Phe23		Carboxylate	Carbon hydrogen bond	3.19
	Leu27		Benzene ring	π-Sigma	3.51
	Val25		Cyclopropyl	Alkyl-alkyl	4.15
	Phe23		Cyclopropyl	π-Alkyl	5.02
	Leu27		Pyridone ring	π-Alkyl	5.15
	Val25		Pyridone ring	π-Alkyl	4.95
	Phe26	Levofloxacin	Carboxylate	π-Anion	3.87
	Val29		Methyl	Alkyl-alkyl	4.30
	Leu27		Pyridone ring	π-Alkyl	5.18
	Val29		Pyridone ring	π-Alkyl	4.82
	Leu27		Benzene ring	π-Alkyl	5.40
	Val25		Benzene ring	π-Alkyl	4.65
	Ala22	Dexamethasone	Cyclohexane ring	Alkyl-alkyl	4.78
	Ala22		Cyclohexane ring	Alkyl-alkyl	4.60
	Ala22		Methyl group	Alkyl-alkyl	4.00
	Ala22		Methyl group	Alkyl-alkyl	3.66
	Phe26		Methyl group	π-Alkyl	4.84
	Phe26		Methyl group	π-Alkyl	4.62
	Phe26		Cyclopentane ring	π-Alkyl	5.38
	Leu19		Methyl group	Alkyl-alkyl	5.17
	Phe26		Methyl group	Alkyl-alkyl	5.48
	Phe23	Chloroquine	Ethyl	Carbon hydrogen bond	3.74
	Phe26		Pyridine ring	π–π t-shaped	4.82
	Phe26		Benzene ring	π–π t-shaped	5.30
	Ala22		Chlorine	Halogen	3.57
	Leu19		Chlorine	Halogen	4.76
	Ala22		Benzene ring	π-Alkyl	4.97

The carboxylate group of levofloxacin was involved in hydrogen bonding with Asp435. In addition, fluorine atom forms another hydrogen bond with Glu389 increases the stability of the complex.

**Fig. 7:**
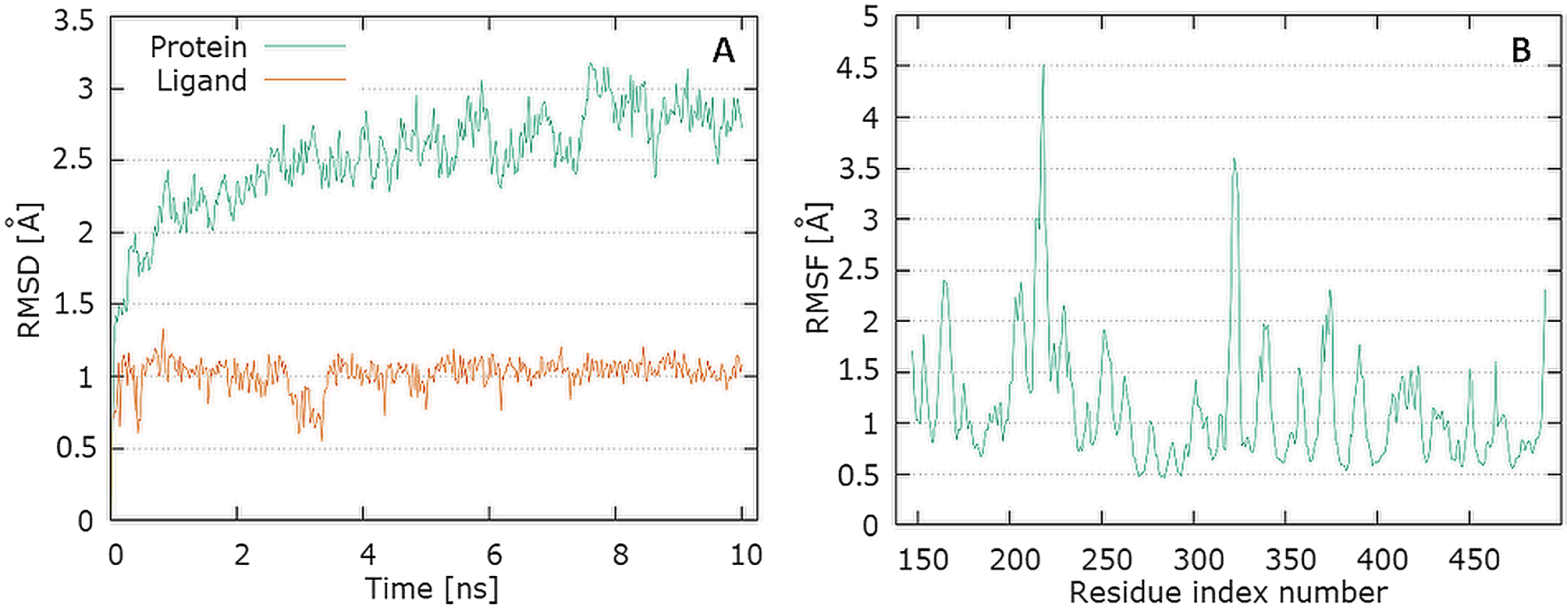
Graphical representation of the plots showing protein CαRMSD [Å] versus time (**A**) and protein RMSF [Å] versus residue index number (**B**) for levofloxacin complex of TMPRSS2 protein

The analysis of the trajectory for TMPRSS2 protein Cα versus time for the simulation with levofloxacin (Fig. 7A) shows that complex attained an equilibrium value of 2.7 Å at around 8 ns. Initially, a sharp increase in the deviation value from 0.5 to 2.5 Å occurs in the first 6 ns. This proves significant protein conformational changes during this period. The plot for protein RMSF versus residue number index shown in Fig. 7B describes fluctuations in the range of 3‒8 Å for complex. The fluctuations in both the terminal ends were higher. The catalytic residues are no longer higher then 1 Å for both complexes, and are suitable for stable binding. The data presented in Fig. 5B show the results of analysis of hydrogen bond contacts in the levofloxacin–TMPRSS2 protein complex. Levofloxacin forms seven hydrogen bonds, and the binding with Glu389 residue occurs in the complex at the 8.2% time of measurement.

**Fig. 8:**
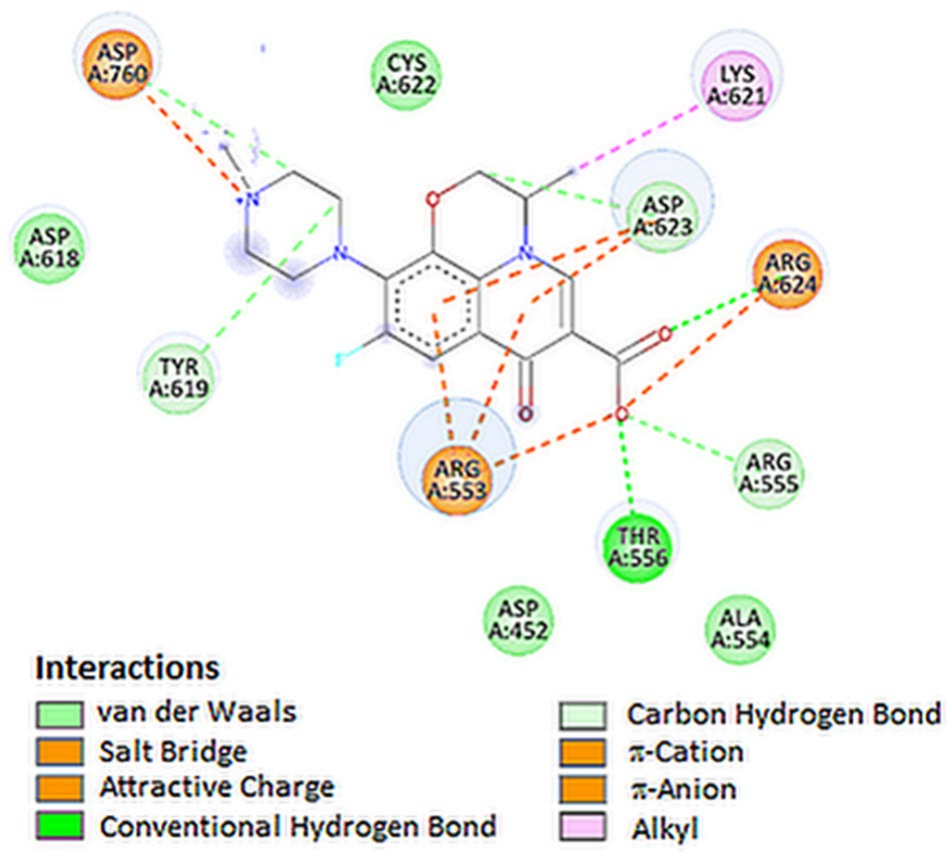
2D visualization of interaction between levofloxacin and RdRp

For coronaviruses, RNA-dependent RNA polymerase (RdRp) is an important enzyme that catalyzes the replication of RNA from RNA templates. RdRp has a huge and deep groove as an active site for the polymerization of RNA which includes Val557 along with the surrounding amino acids [31]. During docking of tested compound to RdRp, the best docked ligands were the dexamethasone and levofloxacin (Figs. 2C and 8). Levofloxacin formed hydrogen bonds with Thr556 and Arg624 and additionally formed salt bridge with Arg553 residue. Moreover, levofloxacin interact with residues of Arg624 and Asp760 forming a attractive charge interactions (Table 3).

**Fig. 9:**
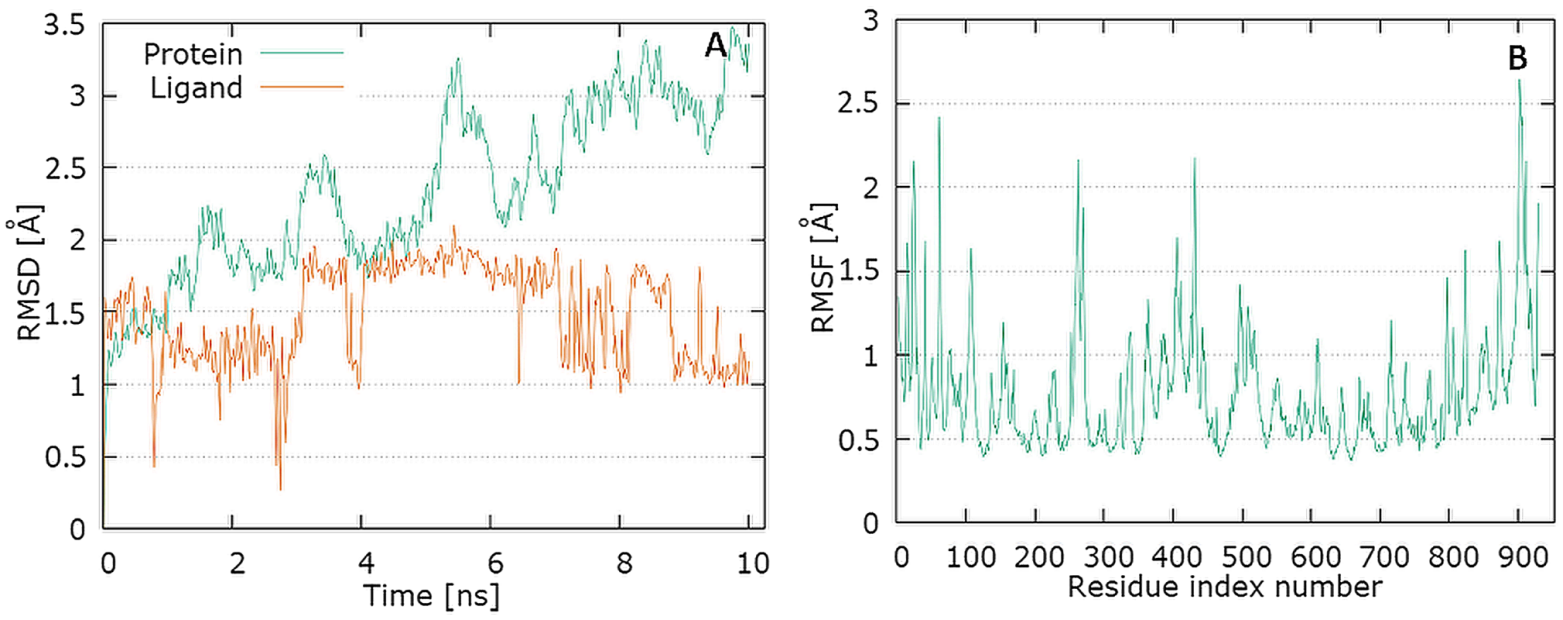
Graphical representation of the plots showing protein CαRMSD [Å] versus time (**A**) and protein RMSF [Å] versus residue index number (**B**) for levofloxacin complex of RdRp

The plots for RdRp protein Cα and the ligand backbone RMSD versus time for the simulation are shown in Fig. 9A. From initial stage, a sharp increase in the deviation value occurs in the 10 ns pointing toward a large conformational change in the protein and ligand during that period.

The plot for protein RMSF versus residue number index shown in Fig. 9B describes fluctuations in the range of 0.5‒2.5 Å for tested complex. The catalytic residues (residues 618–720) are no longer higher then 1 Å and is suitable for stable binding. Results of hydrogen bond interactions in the levofloxacin–RdRp complex are presented In Fig. 5C. They indicate that five hydrogen bonds are formed in this complex during the measurement time.

**Fig. 10:**
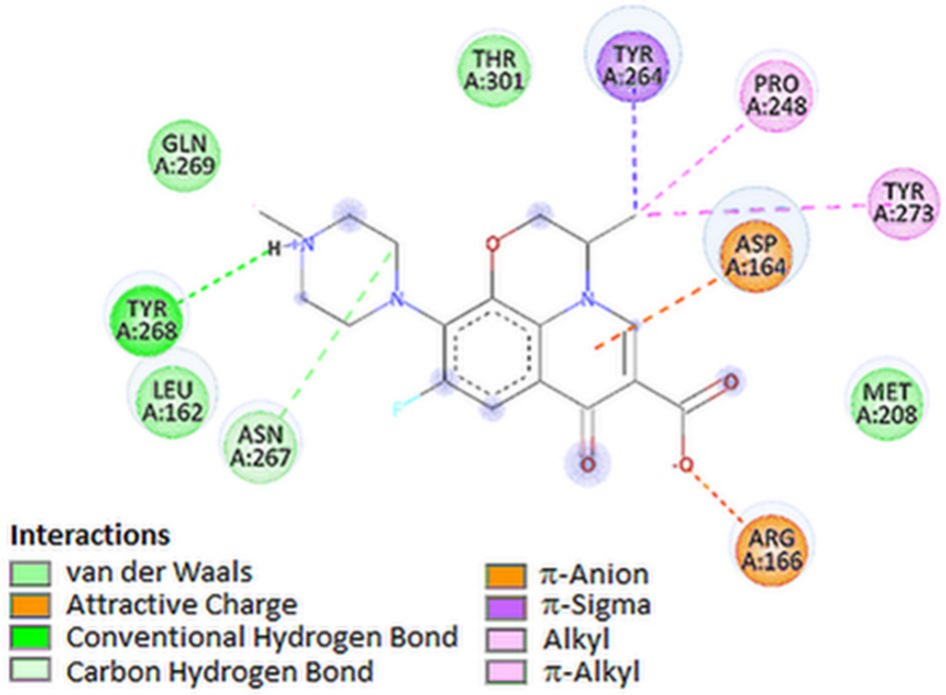
2D visualization of interaction between levofloxacin and PLpro

One of the attractive antiviral drug targets is the SARS-CoV-2 papain-like protease (PLpro). PLpro is responsible for processing three cleavage sites of the viral polyprotein to release mature non-structural proteins 1, 2 and 3. The in vitro studies have also shown that PLpro exhibit two other proteolytic activities: removal of ubiquitin and ubiquitin-like protein ISG15 (interferon-induced gene 15) from cellular proteins. In the case of PLpro, the drug molecules bind to S3/S4 domains. The S3/S4 pocket contained residues Asp164, Val165, Arg166, Glu167, Met 208, Ala246, Pro247, Pro248, Tyr 264, Gly266, Asn267, Tyr 268, Gln269, Cys217, Gly271, Tyr273, Thr301 and Asp302 [32]. According to the results of docking for the target PLpro protein, levofloxacin showed a higher degree of fit in the following order: levofloxacin > dexamethasone > ciprofloxacin > chloroquine (see Table 1). The optimal docking poses of levofloxacin with amino acid residues, inside the active side of PLpro, are presented in Fig. 2D. The top docked pose of levofloxacin in the binding site showed hydrophobic interactions with Asn267, Asp164, Tyr264, Pro248 and Tyr273. The carboxylate group of levofloxacin were involved in attractive charge interaction with Arg166 and protonated nitrogen in piperazine unit was involved in hydrogen bonding with Tyr268 (Figs. 2D, 10 and Table 3).

The PLpro plots for protein Cα versus time for the simulation are shown in Fig. 10A. At Initial stage, a sharp increase in the deviation value from 0.2 to 1.5 Å nm occurs in the first 2 ns pointing toward a large conformational change in the protein during that period. The protein tend to fluctuate between 5 and 7 ns. The RMSD value observed above this limit was quite stable for binding of the ligand with the protein.

**Fig. 11:**
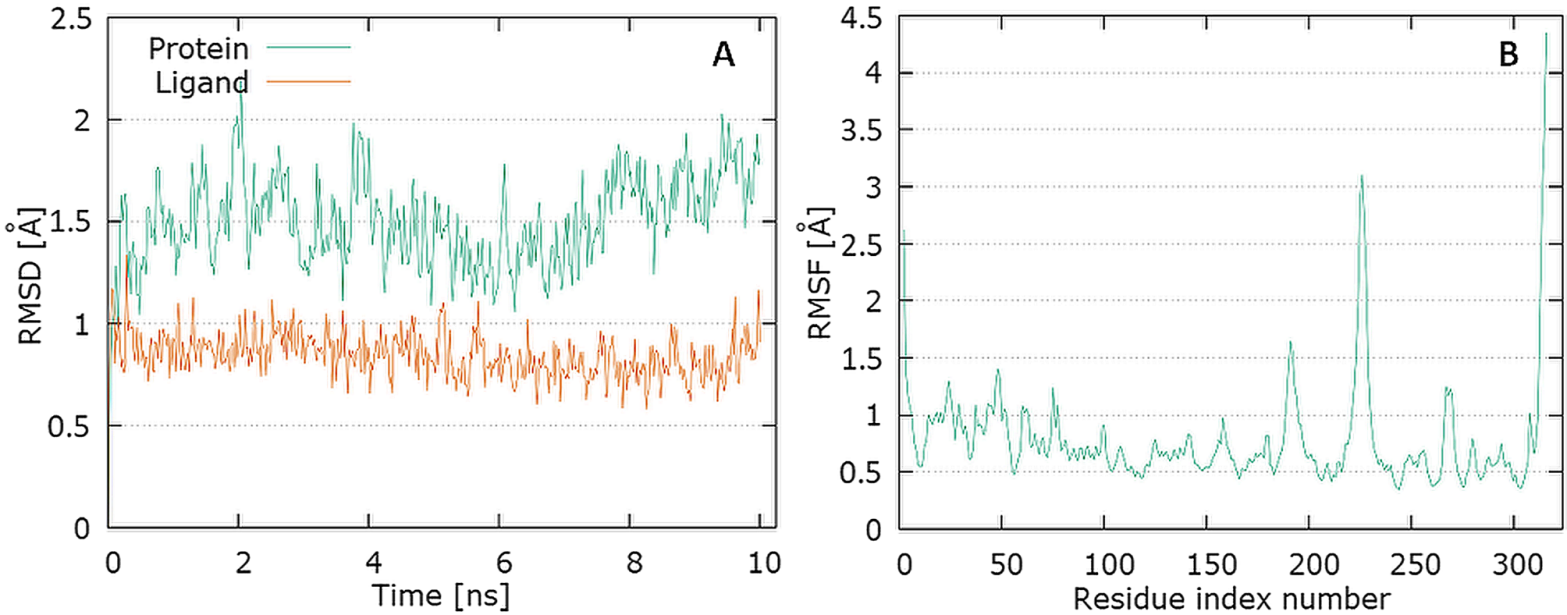
Graphical representation of the plots showing protein CαRMSD [Å] versus time (**A**) and protein RMSF [Å] versus residue index number (**B**) for levofloxacin complex of PLpro

The plot for ligand RMSD shown in Fig. 10A describes fluctuations in the range of 0.6–1.1 Å for levofloxacin and has proved the stability of ligand–protein complex. In Fig. 10B, the plot for protein RMSF versus residue number index shown fluctuations in the range of 0.5‒3 Å for tested complex. The catalytic residues are no longer higher than 1 Å and is suitable for stable binding. The results of the analysis presented in Fig. 5D show that levofloxacin forms four hydrogen bonds with the PL protein (Fig. 11).

**Fig. 12:**
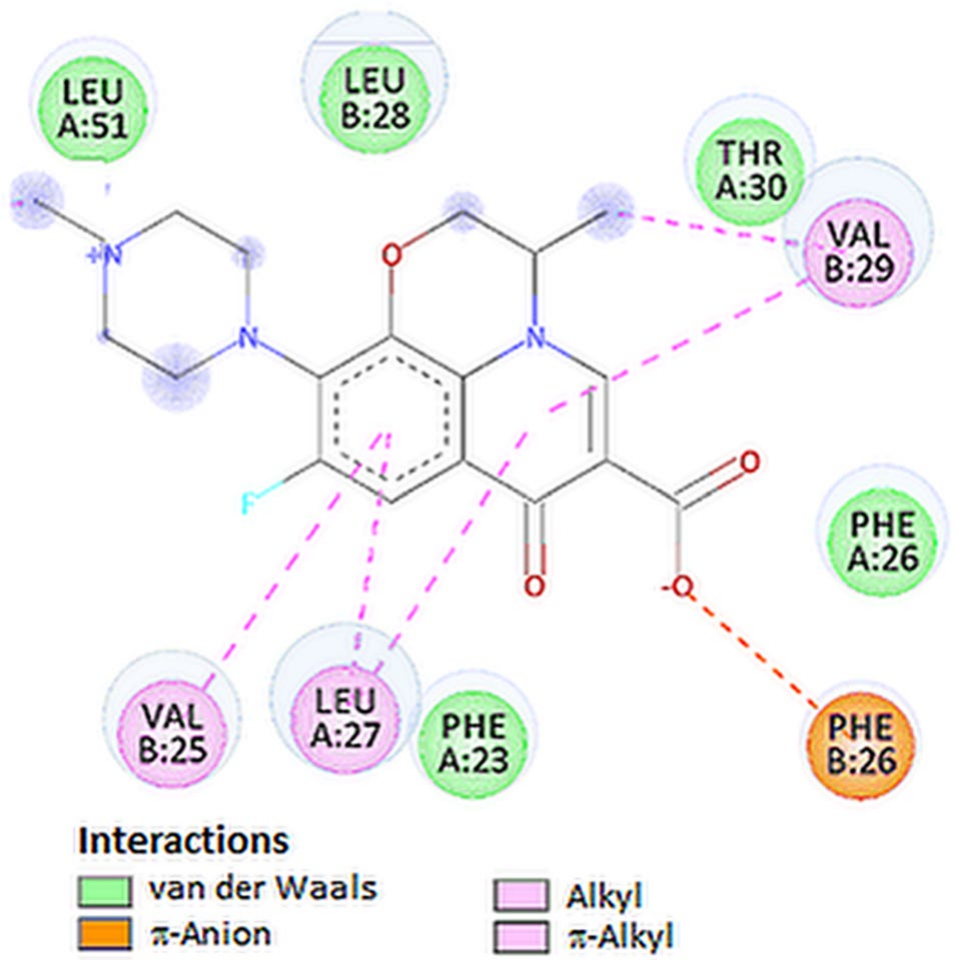
2D visualization of interaction between levofloxacin and E protein

The active site pocket present in the SARS-CoV-2 E protein was obtained from previously published literature [31]. The results revealed that 44 amino acids are involved in the formation of the active site, that is: Glu8, Thr11, Leu12, Val14, Asn15, Val17, Leu18, Leu19, Phe20, Leu21, Ala22, Phe23, Val24, Val25, Phe26, Leu27, Leu28, Val29, Thr30, Leu31, Ala32, Ile33, Leu34, Thr35, Ala36, Leu37, Arg38, Leu39, Ala40, Tyr42, Cys43, Ala44, Ile46, Val47, Val49, Leu51, Pro54, Val56, Tyr57, Ser60, Arg61, Lys63, Asn64, and Leu65. It is pertinent to note that two amino acids, namely Val25 and Phe26, play a key role when interacting with ligands [28, 29]. The tested compounds demonstrate a degree of fit in the following order: levofloxacin = dexamethasone > ciprofloxacin > chloroquine (Table 1). Levofloxacin formed, with the active site of E protein, a network of hydrophobic interactions with amino acid residues in the hinge region area (Figs. 2E and 12). Levofloxacin can reach deep into the hydrophobic pocket region to form interactions with Phe26, Leu27, Val29, Leu27, Ala32 and Val25 amino acid residues (Table 3). As can be seen, the top docked poses of levofloxacin exhibit hydrophobic interactions with the Val25 and Phe26 amino acid residues which play an important role in the regulation of envelope protein activity.

Moreover, levofloxacin interact with residue of Phe26 forming an attractive π-anion interaction.

**Fig. 13:**
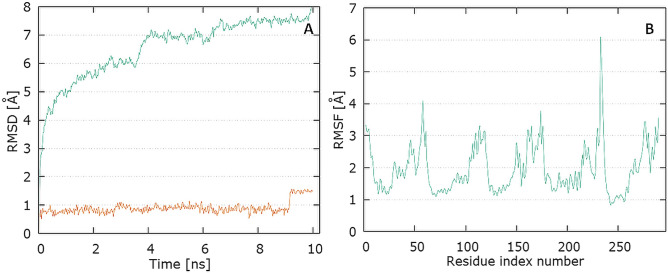
Graphical representation of the plots showing protein CαRMSD (Å) versus time (**A**) and protein RMSF [Å] versus residue index number (**B**) for levofloxacin complex of E protein

The plots for E protein Cα versus time for the simulation are shown in Fig. 13A It shows that complex attained an equilibrium value of 7.5 Å at around 7 ns. Analyzing the levofloxacin backbone RMSD from the plot in Fig. 12 indicates a plateau value of 0.9 Å for the ligand at around 0.2 ns. The deviation value observed was within limits, which is quite stable for binding of the ligand with the protein.

In Fig. 13B, the plot for protein RMSF versus residue number index shown fluctuations in the range of 1‒6 Å for tested complex. The catalytic residues (residue 23–29) are no longer higher than 1 Å and is suitable for stable binding. The results of the analysis presented in Fig. 5E show that levofloxacin forms only one hydrogen bond with the E protein, at the 8.9% time of measurement.

## Discussion

The urgent need for an effective inhibitor of crucial viral proteins is the reason for development of new testing techniques. Recently it was shown by us that ciprofloxacin and moxifloxacin could interact with COVID-19 Main Protease (M^pro^) [14].

In an attempt to test ciprofloxacin and levofloxacin against uncovered SARS-CoV-2 targeted proteins we use molecular docking technique for the probable repurpose of fluoroquinolones towards SARS-CoV-2. The best recorded binding energy value was obtained for ciprofloxacin–S protein complex. Levofloxacin binds strongly to the E- and PL^PRO^ proteins and RNA-dependent RNA polymerase. Molecular dynamics simulations were performed for five best-scoring complexes, for 10 ns and up to 20 ns—in the case of S protein, using NAMD simulation software. RMSD plots were examined to understand the convergence of the molecules over the simulation period. Four complexes: ciprofloxacin-S protein, levofloxacin-E protein, levofloxacin-TMPRSS2 protein and levofloxacin- papain-like protease showed convergence at the end of the simulation period. Results suggest a possible ability of the tested fluoroquinolones for binding to newly emerged viral proteins—S-, E- and TMPRSS2 proteins and papain-like protease (PL^PRO^). Based on previously conducted study [33] it could be noted that a new lead compound (identified via high-throughput screening of a diverse chemical library) and its benzodioxole derivatives could interact (in silico analysis) and therefore inhibit PLpro activity. We believe that both ciprofloxacin and levofloxacin should furthermore be tested, and these drugs in vitro inhibitory potential needs to be investigated by various biochemical assays. However it should be taken into consideration that both ciprofloxacin and levofloxacin were found to suppressed SARS-CoV-2 replication in Vero cells and A549 cells engineered to overexpress ACE2 [34]. Despite the fact, that this effect was demonstrated at high micromolar concentrations in both cell types it cannot be excluded that the tested fluoroquinolone derivatives are not able to achieve such a high concentrations in targeted tissues. Taking into account good pharmacokinetic properties of fluoroquinolone antibiotics, ciprofloxacin is able to reach high concentrations in the target tissues (higher than in plasma), which consist the basis for its use in the treatment of the respiratory and urinary tract infections [35, 36]. In the case of ciprofloxacin, it was noticed that the concentration of the drug after oral administration in the lung tissue may reach the value up to 7 times higher than in the serum [37]. Moreover, it has been documented that fluoroquinolones antiviral potency likely varies by cell type and level of cellular differentiation [38, 39]. In addition, it is possible that synergism with other drugs could improve the antiviral activity of fluoroquinolones to suppress the replication of SARS-CoV-2.

Taking into consideration, that further studies need to be conducted to elucidate the in vitro as well as in vivo efficacy of the tested fluoroquinolone derivatives that could strengthen findings reported in the present study, we want to demonstrate our recent results in anti-SARS-CoV-2 research as soon possible.
